# Role of Artificial Intelligence in Patient Safety Outcomes: Systematic Literature Review

**DOI:** 10.2196/18599

**Published:** 2020-07-24

**Authors:** Avishek Choudhury, Onur Asan

**Affiliations:** 1 School of Systems and Enterprises Stevens Institute of Technology Hoboken, NJ United States

**Keywords:** artificial intelligence, patient safety, drug safety, clinical error, report analysis, natural language processing, drug, review

## Abstract

**Background:**

Artificial intelligence (AI) provides opportunities to identify the health risks of patients and thus influence patient safety outcomes.

**Objective:**

The purpose of this systematic literature review was to identify and analyze quantitative studies utilizing or integrating AI to address and report clinical-level patient safety outcomes.

**Methods:**

We restricted our search to the PubMed, PubMed Central, and Web of Science databases to retrieve research articles published in English between January 2009 and August 2019. We focused on quantitative studies that reported positive, negative, or intermediate changes in patient safety outcomes using AI apps, specifically those based on machine-learning algorithms and natural language processing. Quantitative studies reporting only AI performance but not its influence on patient safety outcomes were excluded from further review.

**Results:**

We identified 53 eligible studies, which were summarized concerning their patient safety subcategories, the most frequently used AI, and reported performance metrics. Recognized safety subcategories were clinical alarms (n=9; mainly based on decision tree models), clinical reports (n=21; based on support vector machine models), and drug safety (n=23; mainly based on decision tree models). Analysis of these 53 studies also identified two essential findings: (1) the lack of a standardized benchmark and (2) heterogeneity in AI reporting.

**Conclusions:**

This systematic review indicates that AI-enabled decision support systems, when implemented correctly, can aid in enhancing patient safety by improving error detection, patient stratification, and drug management. Future work is still needed for robust validation of these systems in prospective and real-world clinical environments to understand how well AI can predict safety outcomes in health care settings.

## Introduction

Patient safety is defined as the absence of preventable harm to a patient and minimization of the risk of harm associated with the health care process [1,2]. Every part of the care-giving process involves a certain degree of inherent risk. Since resolution WHA55.18 on “Quality of Care: Patient Safety” at the 55th World Health Assembly was proposed in 2002, there has been increasing attention paid to patient safety concerns and adverse events in health care settings [3]. Despite the safety initiatives and investments made by federal and local governments, private agencies, and concerned institutions, studies continue to report unfavorable patient safety outcomes [4,5].

The integration of artificial intelligence (AI) into the health care system is not only changing dynamics such as the role of health care providers but is also creating new potential to improve patient safety outcomes [6] and the quality of care [7]. The term AI can be broadly defined as a computer program that is capable of making intelligent decisions [8]. The operational definition of AI we adopt in this review is the ability of a computer or health care device to analyze extensive health care data, reveal hidden knowledge, identify risks, and enhance communication [9]. In this regard, AI encompasses machine learning and natural language processing. Machine learning enables computers to utilize labeled (supervised learning) or unlabeled (unsupervised learning) data to identify latent information or make predictions about the data without explicit programming [9]. Among different types of AI, machine learning and natural language processing specifically have societal impacts in the health care domain [10] and are also frequently used in the health care field [9-12].

**Figure 1:**
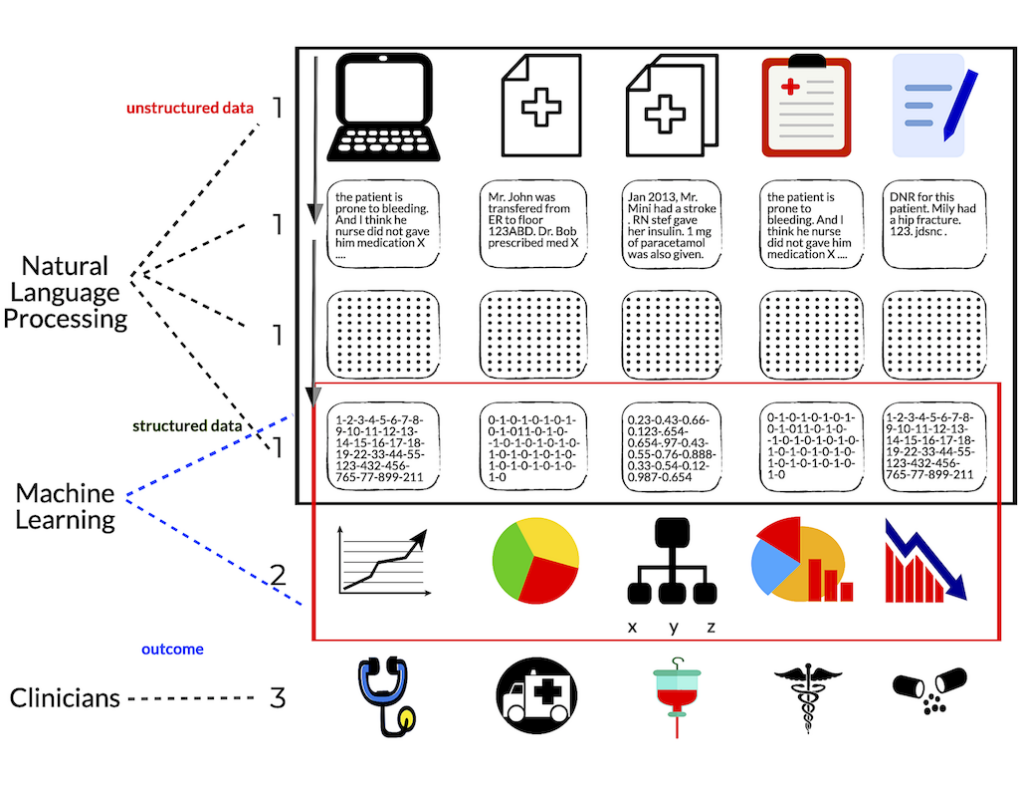
Schematic illustration of how natural language processing converts unstructured text to machine-readable structured data, which can then be analyzed by machine-learning algorithms.

The third category within machine learning is known as reinforcement learning, in which an algorithm attempts to accomplish a task while learning from its successes and failures [9]. Machine learning also encompasses artificial neural networks or deep learning [13]. Natural language processing focuses on building a computer’s ability to understand human language and consecutively transform text to machine-readable structured data, which can then be analyzed by machine-learning techniques [14]. In the literature, the boundary defining natural language processing and machine learning is not clearly defined. However, as illustrated in Figure 1, studies in the field of health care have been using natural language processing in conjunction with machine-learning algorithms [15].

AI has potential to assist clinicians in making better diagnoses [16-18], and has contributed to the fields of drug development [19-21], personalized medicine, and patient care monitoring [14,22-24]. AI has also been embedded in electronic health record (EHR) systems to identify, assess, and mitigate threats to patient safety [25]. However, with the deployment of AI in health care, several risks and challenges can emerge at an individual level (eg, awareness, education, trust), macrolevel (eg, regulation and policies, risk of injuries due to AI errors), and technical level (eg, usability, performance, data privacy and security).

The measure of AI accuracy does not necessarily indicate clinical efficiency [26]. Another common measure, the area under the receiver operating characteristic curve (AUROC), is also not necessarily the best metric for clinical applicability [27]. Such AI metrics might not be easily understood by clinicians or might not be clinically meaningful [28]. Moreover, AI models have been evaluated using a variety of parameters and report different measure(s) such as the *F1* score, accuracy, and false-positive rate, which are indicative of different aspects of AI’s analytical performance. Understanding the functioning of complex AI requires technical knowledge that is not common among clinicians. Moreover, clinicians do not necessarily have the training to identify underlying glitches of the AI, such as data bias, overfitting, or other software errors that might result in misleading outcomes. Such flaws in AI can result in incorrect medication dosage and poor treatment [29-33].

Furthermore, a system error in a widely used AI might lead to mass patient injuries compared to a limited number of patient injuries due to a provider’s error [34]. Additionally, there have been instances where traditional analytical methods outperformed machine-learning techniques [9]. Owing to the wide range of effectiveness of AI, it is crucial to understand both the promising and deterring impacts of AI on patient safety outcomes [35].

AI in the health care system can assist at both the “clinical” and “diagnostic” levels [36]. AI provides a powerful tool that can be implemented within the health care domain to reveal subtle patterns in data, and these patterns can then be interpreted by clinicians to identify new clinical and health-related issues [9]. Recent studies and reviews have primarily focused on the performance of AI at the diagnostic level, such as for disease identification [37-42], and the application of AI robotics in surgery and disease management [43-46]. Other studies have also implemented AI technologies to assist at the clinical level, including assessing fall risks [47] and medication errors [48,49]. However, many of these studies are centered around AI development and performance and there is a notable lack of studies reviewing the role and impact of AI used at the clinical level on patient safety outcomes.

Many studies have reported high accuracy of AI in health care. However, its actual influence (negative or positive) can only be realized when it is integrated into clinical settings or interpreted and used by care providers [50]. Therefore, in our view, patient safety and AI performance might not necessarily complement each other. AI in health care depends on data sources such as EHR systems, sensor data, and patient-reported data. EHR systems may contain more severe cases for specific patient populations. Certain patient populations may have more ailments or may be seen at multiple institutions. Certain subgroups of patients with rare diseases may not exist in sufficient numbers for a predictive analytic algorithm. Thus, clinical data retrieved from EHRs might be prone to biases [9,50]. Owing to these potential biases, AI accuracy might be misleading [51] when trained on a small subgroup or small sample size of patients with rare ailments.

Furthermore, patients with limited access to health care may receive fewer diagnostic tests and medications and may have insufficient health information in the EHR to trigger an early intervention [52]. In addition, institutions record patient information differently; as a result, if AI models trained at one institution are implemented to analyze data at another institution, this may result in errors [52]. For instance, machine-learning algorithms developed at a university hospital to predict patient-reported outcome measures, which tend to be documented by patients who have high education as well as high income, may not be applicable when implemented at a community hospital that primarily serves underrepresented patient groups with low income.

**Figure 2:**
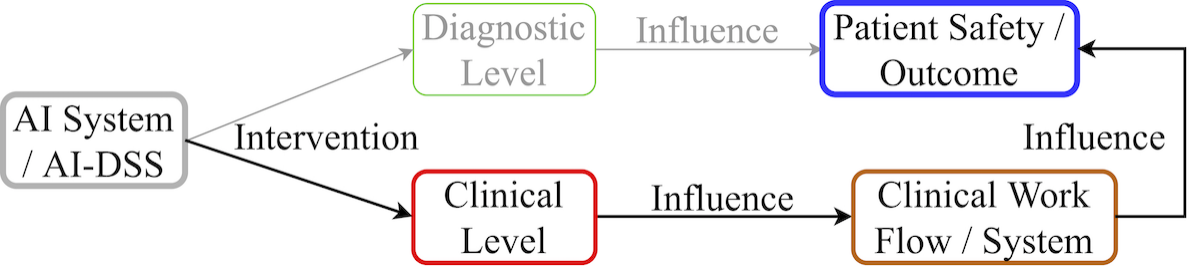
Artificial intelligence (AI) route to patient safety via “Clinical” and “Diagnostic” level interventions. DSS: decision support system.

A review [53] conducted in 2017 reported that only about 54% of studies that developed prediction models based on EHRs accounted for missing data. Recent studies and reviews have been primarily focusing on the performance and influence of AI systems at a diagnostic level, such as for disease identification [37-42], and the influence of AI robotics in surgery and disease management [43-46]; however, there is a lack of studies reviewing and reporting the impact of AI used at the clinical level on patient safety outcomes, as well as characteristics of the AI algorithms used. Thus, it is essential to study how AI has been shown to influence patient safety outcomes at the clinical level, along with reported AI performance in the literature. In this systematic review, we address this gap by exploring the studies that utilized AI algorithms as defined in this review to address and report changes in patient safety outcomes at the clinical level (Figure 2).

## Methods

### Protocol Registration

This systematic review is reported according to the Preferred Reporting Items for Systematic Reviews and Meta-Analysis (PRISMA) guidelines [54]. We followed the PRISMA Checklist (see Multimedia Appendix 1). Our protocol [55] was registered with the Open Science Framework on September 15, 2019.

### Information Sources

We searched for peer-reviewed publications in the PubMed, PubMed Central, and Web of Science databases from January 2009 to August 2019 to identify articles within the scope and eligibility criteria of this systematic literature review.

### Search Strategy

We followed a systematic approach of creating all search terms to capture all related and eligible papers in the searched databases. Keywords used in the search were initially determined by a preliminary review of the literature and then modified based on feedback from content experts as well as our institution’s librarian.

We then refined the search strategy in collaboration with the librarian to ensure that all clinical-level patient safety-related papers (as shown in Figure 2) were covered in our review and determined the Medical Subject Headings (MeSH) terms. We grouped the query keywords, which were derived from MeSH terms and combined through Boolean (AND/OR) operators to identify all relevant studies that matched with our scope and inclusion criteria.

**Figure 3:**
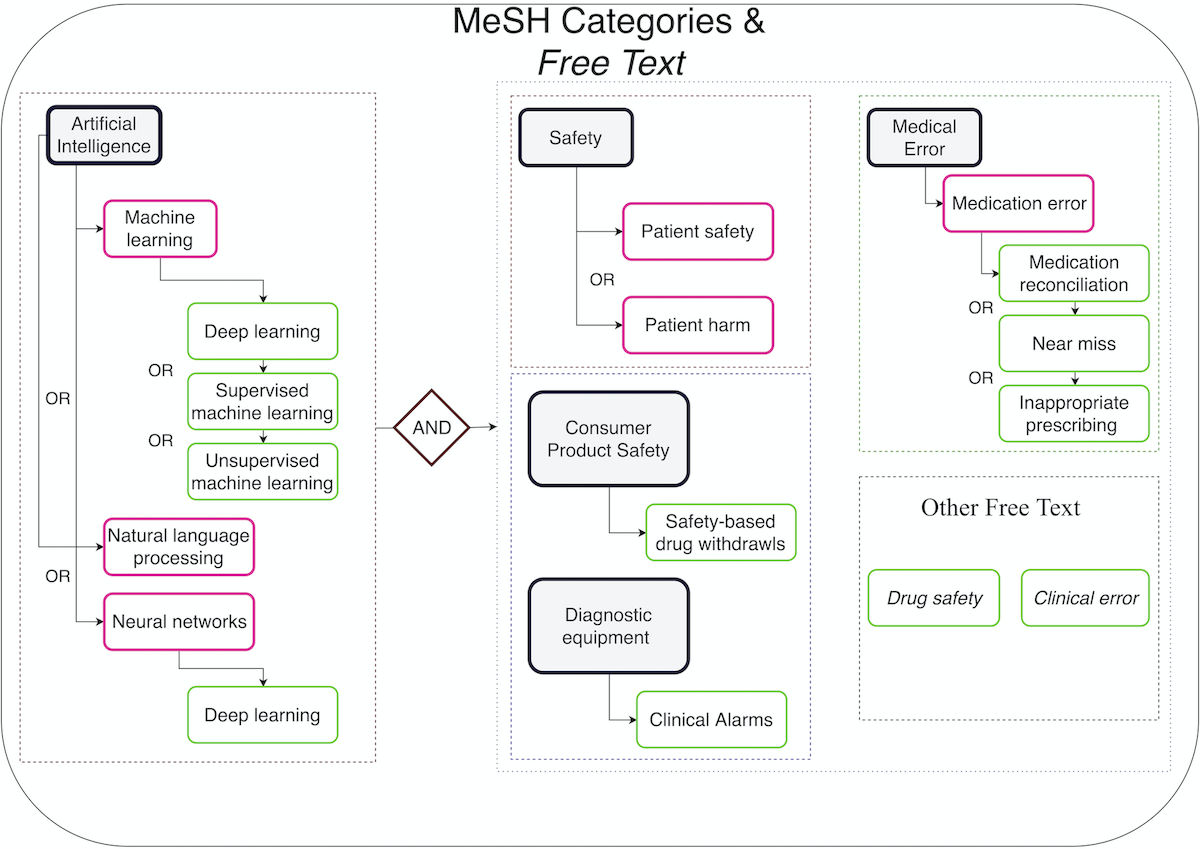
Medical Subject Heading (MeSH) terms and free text used in the systematic literature review.

The keywords consisted of MeSH terms such as “safety [MeSH]” and “artificial intelligence [MeSH],” in combination with narrower MeSH terms (subheadings/related words/phrases) and free text for “artificial intelligence” and “safety.” We also included broader key terms to encompass all latent risk factors affecting patient safety. The final search keywords (Figure 3) described below were used to explore all databases.

MeSH terms are organized in a tree-like hierarchy, with more specific (narrower) terms arranged beneath broader terms. By default, PubMed includes all of the narrow items in the search in a strategy known as “exploding” the MeSH term [56]. Moreover, the inclusion of MeSH terms optimizes the search strategy [56]. Therefore, the final search query for PubMed was as follows: (“patient safety” OR “safety” [MeSH] OR “drug safety” OR “safety-based Drug withdraws” [MeSH] OR “medication error” OR “Medication Error” [MeSH] OR “medication reconciliation” OR “near miss” OR “inappropriate prescribing” OR “clinical error” OR “Clinical alarms” [MeSH]) AND (“Machine learning” [MeSH] OR “Machine learning” OR “Deep learning” [MeSH] OR “Deep learning” OR “natural language processing” [MeSH] OR “natural language processing”).

### Inclusion and Exclusion Criteria

This study focused on peer-reviewed publications satisfying the following two primary conditions: (1) implementation of machine-learning or natural language processing techniques to address patient safety concerns, and (2) discussing or reporting the impact or changes in clinical-level patient safety outcomes. Any papers that failed to satisfy both conditions were excluded from this review. For instance, studies only focusing on developing or evaluating machine-learning models that did not report or discuss changes or impact on clinical-level patient safety outcomes were excluded, as well as studies that used AI beyond our scopes, such as robotics or computer vision. Secondary research such as reviews, commentaries, and conceptual articles was excluded from this study. The search was restricted to papers published in English between January 2009 and August 2019.

### Study Selection and Quality Assurance

The two authors together reviewed all of the retrieved publications for eligibility. We first screened the publications by studying the titles and abstracts and removed duplications. We then read the full text for the remaining papers and finalized the selection. To minimize any selection bias, all discrepancies were resolved by discussion requiring consensus from both reviewers and the librarian. Before finalizing the list of papers, we consulted our results and searched keywords with the librarian to ensure that no relevant articles were missed.

A data abstraction form was used to record standardized information from each paper as follows: authors, aims, objectives of the study, methods, and findings. Using this form, we categorized each article based on the type of AI algorithm as well as clinical-level patient safety outcomes reported.

## Results

### Study Selection

**Figure 4:**
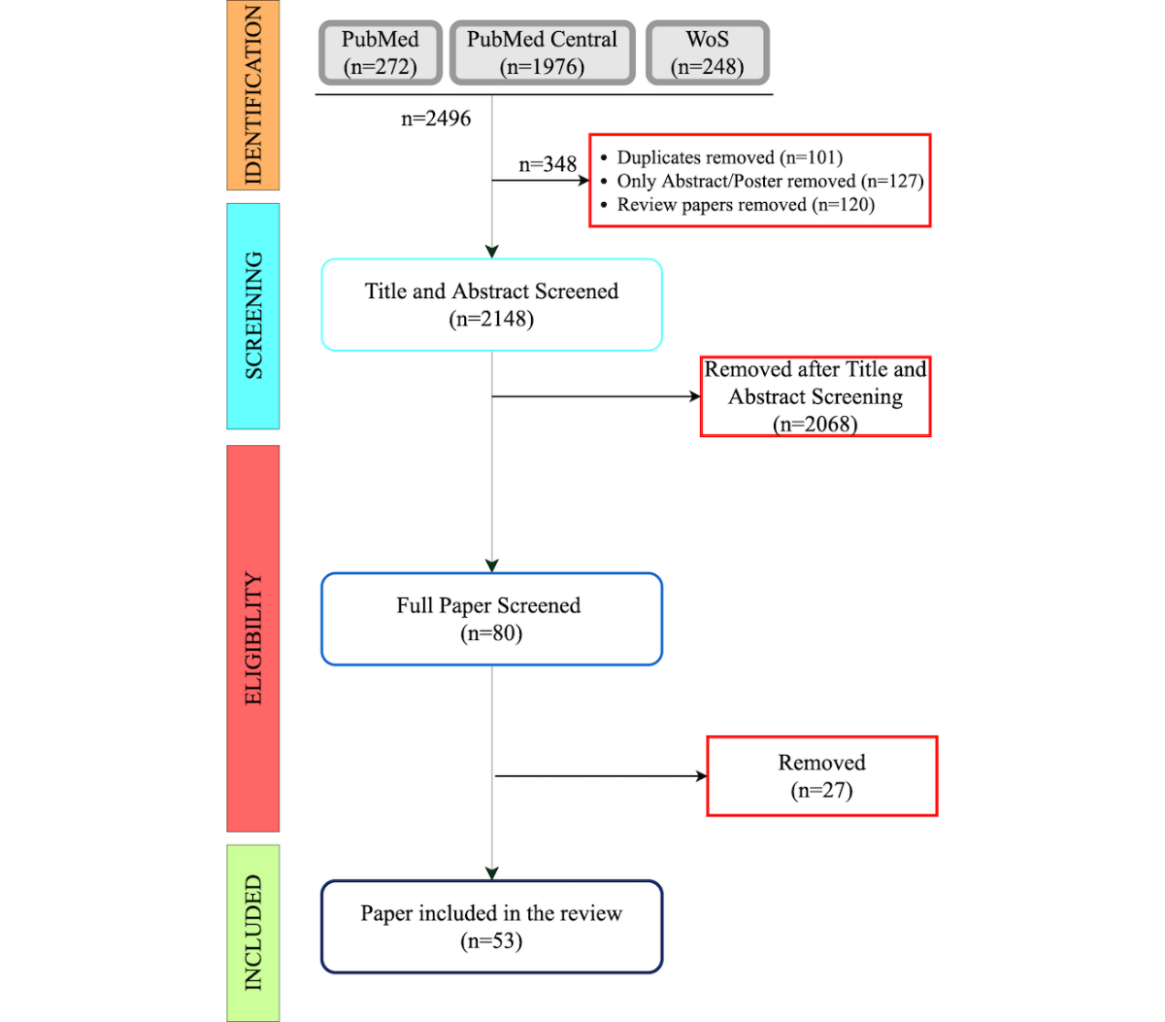
Preferred Reporting Items for Systematic Reviews and Meta-Analysis (PRISMA) flow chart illustrating the process of selecting eligible publications for inclusion in the systematic review. WoS: Web of Science.

Figure 4 illustrates the flowchart of the selection process of the articles included in this systematic literature review. The initial search using a set of queries returned 272 publications in PubMed, 1976 publications in PubMed Central, and 248 publications in Web of Science for a total of 2496 articles. We used EndNote X9.3.2 to manage the filtering and duplication removal process. As a first step, we removed duplicates (n=101), all review/opinion/perspective papers (n=120), and posters or short abstracts (n=127). The two authors then applied a second filtering step by reading abstracts and titles (n=2148). The screening process followed the inclusion and exclusion criteria explained above, resulting in 80 papers eligible for a full-text review. The authors then removed 27 more articles based on the full-text review. Hence, the final number of studies included in the systematic review was 53, with consensus from both authors.

Table 1 outlines all characteristics of the final selected studies (n=53), including the objective of the study and AI methods used, as well as classification of all articles by latent risk factors of patient safety according to (a) Clinical Alarms/Alerts, (b) Clinical Reports, and (c) Adverse Drug Event/Drug Safety. Table 1 also reports the findings obtained regarding changes in patient safety outcomes.

The studies mostly reported positive changes in patient safety outcomes, and in most cases improved or outperformed traditional methods. For instance, AI was successful in minimizing false alarms in several studies and also improved real-time safety reporting systems (Table 1). AI was also able to extract useful information from clinical reports. For example, AI helped in classifying patients based on their ailments and severity, identified common incidents such as fall risks, delivery delays, hospital information technology errors, bleeding complications, and others that pose risks to patient safety. AI also helped in minimizing adverse drug effects. Further, some studies reported poor outcomes of AI, in which AI’s classification accuracy was lower than that of clinicians or existing standards.

Table 2 outlines the performance and accuracy measures of AI models used by the final selected studies, demonstrating the heterogeneity in AI performance measures adopted by different studies.

**Table 1:** Evidentiary table of 53 selected publications.

Reference	Objective	Study theme	AI^a^ method	Findings (patient safety outcomes)
Chen et al [57]	To classify alerts as real or artifacts in online noninvasive vital sign data streams and minimize alarm fatigue and missed true instability	Clinical alarms/alerts	KNN^b^, NB^c^, LR^d^, SVM^e^, RF^f^	Machine-learning (ML) models could distinguish clinically relevant pulse arterial O_2_ saturation, blood pressure, and respiratory rate from artifacts in an online monitoring dataset (AUC^g^>0.87)
Ansari et al [58]	To minimize false alarms in the ICU^h^	Clinical alarms/alerts	MMD^i^, DT^j^	ML algorithm along with MMD was effective in suppressing false alarms
Zhang et al [59]	To minimize the rate of false critical arrhythmia alarms	Clinical alarms/alerts	SVM	SVM reduced false alarm rates. The model gave an overall true positive rate of 95% and true negative rate of 85%
Antink et al [60]	To reduce false alarms by using multimodal cardiac signals recorded from a patient monitor	Clinical alarms/alerts	BCT^k^, SVM, RF, RDAC^l^	A false alarm reduction score of 65.52 was achieved; employing an alarm-specific strategy, the model performed at a true positive rate of 95% and true negative rate of 78%. False alarms for extreme tachycardia were suppressed with 100% sensitivity and specificity
Eerikäinen et al [61]	To classify true and false cardiac arrhythmia alarms	Clinical alarms or alerts	RF	Out of 5 false alarms, 4 were suppressed; 77.39% real-time model accuracy
Menard et al [62]	Develop a predictive model that enables Roche/Genentech Quality Program Leads oversight of adverse event reporting at the program, study, site, and patient level.	Clinical alarms/alerts	ML (not disclosed)	The ML method identified the sites by risk of underreporting and enabled real-time safety reporting. The proposed model had an AUC of 0.62, 0.79, and 0.92 for simulation scenarios of 25%, 50%, and 75%, respectively. This project was part of a broader effort at Roche/Genentech Product Development Quality to apply advanced analytics to augment and complement traditional clinical quality assurance approaches
Segal et al [63]	To determine the clinical usefulness of medication error alerts in a real-life inpatient setting	Clinical alarms or alerts	Probabilistic ML	85% of the alerts were clinically valid, and 80% were considered clinically useful; 43% of the alerts caused changes in subsequent medical orders. Thus, the model detected medication errors
Hu et al [64]	To detect clinical deterioration	Clinical alarms or alerts	NN^m^	NN-based model could detect health deterioration such as heart rate variability with more accuracy than one of the best-performing early warning scores (ViEWS). The positive prediction value of NN was 77.58% and the negative prediction value was 99.19%
Kwon et al [65]	To develop alarm systems that predict cardiac arrest early	Clinical alarms or alerts	RF, LR, DEWS^n^, and MEWS^o^	The DEWS identified more than 50% of patients with in-hospital cardiac arrest 14 hours before the event. It allowed medical staff to have enough time to intervene. The AUC and AUPRC^p^ of DEWS was 0.85 and 0.04, respectively, and outperformed MEWS with AUC and AUPC of 0.60 and 0.003, respectively; RF with AUC and AUPC of 0.78 and 0.01, respectively; and LR with AUC and AUPRC of 0.61 and 0.007, respectively. DEWS reduced the number of alarms by 82.2%, 13.5%, and 42.1% compared with the other models at the same sensitivity
Gupta and Patrick [66]	To classify clinical incidents	Clinical Report	J48^q^, NB multinomial, and SVM	The selected models performed poorly in classifying incident categories (48.77% best, using J48), but performed comparatively better in classifying free text (76.49% using NB).
Wang et al [67]	To identify multiple incident types from a single report	Clinical Report	Compares binary relevance, CC^r^	Binary classifier improved identification of common incident types: falls, medications, pressure injury, aggression, documentation problem, and others. Automated identification enabled safety problems to be detected and addressed in a more timely manner
Zhou et al [49]	To extract information from clinical reports	Clinical Report	SVM, NB, RF, and MLP^s^	ML algorithms identified the medication event originating stages, event types, and causes, respectively. The models improved the efficiency of analyzing the medication event reports and learning from the reports in a timely manner with (SVM) *F1* of 0.792 and (RF) *F1* of 0.925
Fong et al [68]	To analyze patient safety reports	Clinical Report	NLP^t^	Pyxis Discrepancy and Pharmacy Delivery Delay were found to be the main two factors affecting patient safety. The NLP models significantly reduced the time required to analyze safety reports
El Messiry et al [69]	To analyze patient feedback	Clinical Report	NLP	Care-related complaints were influenced by money and emotion
Chondrogiannis et al [70]	To identify the meaning of abbreviations used in clinical studies	Clinical Report	NLP	Each clinical study document contained about 6.8 abbreviations. Each abbreviation can have 1.25 meanings on average. This helped in identification of acronyms
Liang and Gong [71]	To extract information from patient safety reports	Clinical Report	Multilabel classification methods	Binary relevance was the best problem transformation algorithm in the multilabeled classifiers. It provided suggestions on how to implement automated classification of patient safety reports in clinical settings
Ong et al [72]	To identify risk events in clinical incident reports	Clinical report	Text classifiers based on SVM	SVM performed well on datasets with diverse incident types (85.8%) and data with patient misidentification (96.4%). About 90% of false positives were found in “near-misses” and 70% of false negative occurred due to spelling errors
Taggart et al [73]	To identify bleeding events using in clinical notes	Clinical Report	NLP, SVM, CNN^u^, and ET^v^	Rule-based NLP was better than the ML approach. NLP detected bleeding complications with 84.6% specificity, 62.7% positive predictive value, and 97.1% negative predictive value. It can thus be used for quality improvement and prevention programs
Denecke et al [74]	To minimize any loss of information during a doctor-patient conversation	Clinical Report	NLP	Electronic health platform provides an intuitive conversational user interface that patients use to connect to their therapist and self-anamnesis app. The app also allows data sharing among treating therapists
Evans et al [75]	To determine the incident type and the severity of harm outcome	Clinical Report	J48, SVM, and NB	The SVM classifier improved the identification of patient safety incidents. Incident reports containing deaths were most easily classified with an accuracy of 72.82%. The severity classifier was not accurate to replace manual scrutiny
Wang et al [76]	To identify the type and severity of patient safety incident reports	Clinical Report	CNN and SVM ensemble	CNN achieved high *F* scores (>85%) across all test datasets when identifying common incident types, including falls, medications, pressure injury, and aggression. It improved the process by 11.9% to 45.10% across different datasets
Klock et al [47]	To understand the root causes of falls and increase learning from fall reports for better prevention of patient falls.	Clinical Report	SVM, RF, and RNN^w^	The model identified high and low scoring fall reports. Most of the patient fall reports scores were between 0.3 and 0.4, indicating poor quality of reports
Li et al [77]	To stratify patient safety adverse event risk and predict safety problems of individual patients	Clinical Report	Ensemble-ML	The adverse event risk score at the 0.1 level could identify 57.2% of adverse events with 26.3% accuracy from 9.2% of the validation sample. The adverse event risk score of 0.04 could identify 85.5% of adverse events
Murff et al [78]	To identify postoperative surgical complications within a comprehensive electronic medical record	Clinical Report	NLP	NLP identified 82% of acute renal failure cases compared with 38% for patient safety indicators. Similar results were obtained for venous thromboembolism (59% vs 46%), pneumonia (64% vs 5%), sepsis (89% vs 34%), and postoperative myocardial infarction (91% vs 89%)
Wang et al [79]	To automate the identification of patient safety incidents in hospitals	Clinical Report	Text-based classifier: LR, SVM	For severity level, the *F* score for severity assessment code (SAC) 1 (extreme risk) was 87.3 and 64% for SAC4 (low risk) on balanced data. With stratified data, a high recall was achieved for SAC1 (82.8%-84%), but precision was poor (6.8%-11.2%). High-risk incidents (SAC2) and medium-risk incidents (SAC3) were often misclassified. Reports about falls, medications, pressure injury, aggression, and blood tests were identified with high recall and precision
Rosenbaum and Baron [80]	To detect Wrong Blood in Tube errors and mitigate patient harm	Clinical Report	LR, SVM	In contrast to the univariate analysis, the best performing multivariate delta check model (SVM) identified errors with a high degree of accuracy (0.97)
McKnight [81]	To improve the ability to extract clinical information from patient safety reports efficiently	Clinical Report	NLP	The semisupervised model categorized patient safety reports into their appropriate patient safety topic and avoided overlaps; 85% of unlabeled reports were assigned correct labels. It helped NCPS^x^ analysts to develop policy and mitigation decisions
Marella et al [82]	To analyze patient safety reports describing health hazards from electronic health records	Clinical Report	Text mining based on: NB, KNN, rule induction	The NB kernel performed best, with an AUC of 0.927, accuracy of 0.855, and *F* score of 0.877. The overall proportion of cases found relevant was comparable between manually and automatically screened cases; 334 reports identified by the model as relevant were identified as not relevant, implying a false-positive rate of 13%. Manual screening identified 4 incorrect predictions, implying a false-negative rate of 29%
Ye et al [83]	To validate a real-time early warning system to predict patients at high risk of inpatient mortality during their hospital episodes	Clinical Report	RF, XGB^y^, boosting SVM, LASSO^z^, and KNN	The modified early warning system accurately predicted the possibility of death for the top 13.3% (34/255) of patients at least 40.8 hours before death
Fong et al [84]	To identify health information technology-related events from patient safety reports	Clinical Report	Unigram and Bigram LR, SVM	Unigram models performed better than Bigram and combined models. It identified HIT^aa^-related events trained on PSE^bb^ free-text descriptions from multiple states and health care systems. The unigram LR model gave an AUC of 0.931 and an *F1* score of 0.765. LR also showed potential to maintain a faster runtime when more reports are analyzed. The final HIT model had less complexity and was more easily sharable
Simon et al [85]	To establish whether patients with type 2 diabetes can safely use PANDIT^cc^ and whether its insulin dosing advice is clinically safe	Drug safety	PANDIT	27 out of 74 (36.5%) PANDIT advice differed from those provided by diabetes nurses. However, only one of these (1.4%) was considered unsafe by the panel
Song et al [86]	To predict drug-drug interactions	Drug safety	SVM	The 10‐fold crossvalidation improved the identification of drug-drug interaction with AUC>0.97, which is significantly greater than the analogously developed ML model (0.67)
Hammann et al [87]	To identify drugs that could be suspected of causing adverse reactions in the central nervous system, liver, and kidneys	Drug safety	CHAID^dd^ and CART^ee^	CART exhibited high predictive accuracy of 78.94% for allergic reactions, 88.69% for renal, and 90.22% for the liver. CHAID model showed a high accuracy of 89.74% for the central nervous system
Bean et al [88]	To predict adverse drug reactions	Drug safety	LR, SVM, DT, NLP, own model	The proposed model (own model) outperformed traditional LR, SVM, DT, and predicted adverse drug reactions with an AUC of 0.92
Hu et al [89]	To predict the appropriateness of initial digoxin dosage and minimize drug-drug adverse interactions	Drug safety	C4.5, KNN, CART, RF, MLP, and LR	In the non drug-drug interaction group, the AUC of RF, MLP, CART, and C4.5 was 0.91, 0.81, 0.79, and 0.784, respectively; for the drug-drug interaction group, the AUC of RF, CART, MLP, and C4.5 was 0.89, 0.79, 0.77, and 0.77, respectively. DT-based approaches and MLP can determine the initial dosage of a high-alert digoxin medication, which can increase drug safety in clinical practice
Tang et al [90]	To identify adverse drug effects from unstructured hospital discharge summaries	Drug safety	NLP	A total of 33 trial sets were evaluated by the algorithm and reviewed by pharmacovigilance experts. After every 6 trial sets, drug and adverse event dictionaries were updated, and rules were modified to improve the system. The model identified adverse events with 92% precision and recall
Hu et al [91]	To predict the dosage of warfarin	Drug safety	KNN, SVR^ff^, NN-BP^gg^, MT^hh^	The proposed model improved warfarin dosage when compared to the baseline (mean absolute error 0.394); reduced mean absolute error by 40.04%
Hasan et al [92]	To improve medication reconciliation task	Drug safety	LR, KNN	Collaborative filtering identified the top 10 missing drugs about 40% to 50% of the time and the therapeutic missing drugs about 50% to 65% of the time
Labovitz et al [93]	To evaluate the use of a mobile AI platform on medication adherence in stroke patients on anticoagulation therapy	Drug safety	Cell phone–based AI platform	Mean (SD) cumulative adherence based on the AI platform was 90.5% (7.5%). Plasma drug concentration levels indicated that adherence was 100% (15/15) and 50% (6/12) in the intervention and control groups, respectively
Long et al [94]	To improve the reconciliation method	Drug safety	iPad-based software tool with an AI algorithm	All patients completed the task. The software improved reconciliation; all patients identified at least one error in their electronic medical record medication list; 8 of 10 patients reported that they would use the device in the future. The entire team (clinical and patients) liked the device and preferred to use it in the future
Reddy et al [95]	To assess proof of concept, safety, and feasibility of ABC4D^ii^ in a free-living environment over 6 weeks	Drug safety	ABC4D	ABC4D was safe for use as an insulin bolus dosing system. A trend suggesting a reduction in postprandial hypoglycemia was observed. The median (IQR) number of postprandial hypoglycemia episodes within 6 h after the meal was 4.5 (2.0-8.2) in week 1 versus 2.0 (0.5-6.5) in week 6 (*P*=.10). No episodes of severe hypoglycemia occurred during the study
Schiff et al [96]	To evaluate the performance and clinical usefulness of medication error alerts generated by an alerting system	Drug safety	MedAware, probabilistic ML	75% of the chart-reviewed alerts generated by MedAware were valid from which medication errors were identified. Of these valid alerts, 75.0% were clinically useful in flagging potential medication errors.
Li et al [97]	To develop a computerized algorithm for medication discrepancy detection and assess its performance on real-world medication reconciliation data	Drug safety	Hybrid system consisting of ML algorithms and NLP	The hybrid algorithm yielded precision (P) of 95.0%, recall (R) of 91.6%, and *F* value of 93.3% on medication entity identification, and *P*=98.7%, R=99.4%, and *F*=99.1% on attribute linkage. The combination of the hybrid system and medication matching system gave *P*=92.4%, R=90.7%, and *F*=91.5%, and *P*=71.5%, R= 65.2%, and *F*=68.2% on classifying the matched and the discrepant medications, respectively
Carrell et al [98]	To identify evidence of problem opioid use in electronic health records	Drug safety	NLP	The NLP-assisted manual review identified an additional 728 (3.1%) patients with evidence of clinically diagnosed problem opioid use in clinical notes.
Tinoco et al [99]	To evaluate the source of information affecting different adverse events	Drug safety	CSS^jj^ (ML)	CSS detected more hospital-associated infections than manual chart review (92% vs 34%); CSS missed events that were not stored in a coded format
Onay et al [100]	To classify approved drugs from withdrawn drugs and thus reduce adverse drug effects	Drug safety	SVM, Boosted and Bagged trees (Ensemble)	The Gaussian SVM model yielded 78% prediction accuracy for the drug dataset, including all diseases. The ensemble of bagged tree and linear SVM models involved 89% of the accuracies for psycholeptics and psychoanalytic drugs
Cai et al [101]	To discover drug-drug interactions from the Food and Drug Administration’s adverse event reporting system and thus prevent patient harm	Drug safety	Causal Association Rule Discovery (CARD)	CARD demonstrated higher accuracy in identifying known drug interactions compared to the traditional method (20% vs 10%); CARD yielded a lower number of drug combinations that are unknown to interact (50% for CARD vs 79% for association rule mining).
Dandala et al [102]	To extract adverse drug events from clinical narratives and automate pharmacovigilance.	Drug safety	BilSTM^kk^, CRF-NN^ll^	Joint modeling improved the identification of adverse drug events from 0.62 to 0.65
Dey et al [103]	To predict and prevent adverse drug reactions at an early stage to enhance drug safety	Drug safety	Deep learning	Neural fingerprints from the deep learning model (AUC=0.72) outperformed all other methods in predicting adverse drug reactions. The model identified important molecular substructures that are associated with specific adverse drug reactions
Yang et al [104]	To identify medications, adverse drug effects, and their relations with clinical notes	Drug safety	MADEx, LSTM-RNN^mm^, CRF^nn^, SVM, RF	MADEx achieved the top-three best performances (*F1* score of 0.8233) for clinical name entity recognition, adverse drug effect, and relations from clinical texts, which outperformed traditional methods
Chapman et al [105]	To identify adverse drug effect symptoms and drugs in clinical notes	Drug safety	NLP	The micro-averaged *F1* score was 80.9% for named entity recognition, 88.1% for relation extraction, and 61.2% for the integrated systems
Lian et al [106]	To detect adverse drug reactions	Drug safety	LRM^oo^, BNM^pp^, BCP-NN^qq^	Experimental results showed the usefulness of the proposed pattern discovery method by improving the standard baseline adverse drug reaction by 23.83%
Huang et al [107]	To predict adverse drug effects	Drug safety	SVM, LR	The proposed computational framework showed that an in silico model built on this framework can achieve satisfactory cardiotoxicity adverse drug reaction prediction performance (median AUC=0.771, accuracy=0.675, sensitivity=0.632, and specificity=0.789).

^a^AI: artificial intelligence.

^b^KNN: K-nearest neighbor.

^c^NB: naive Bayes.

^d^LR: logistic regression.

^e^SVM: support vector machine.

^f^RF: random forest.

^g^AUC: area under the curve.

^h^ICU: intensive care unit.

^i^MMD: multimodal section.

^j^DT: decision tree.

^k^BCT: binary classification tree.

^l^RDAC: regularized discriminant analysis classifier.

^m^NN: neural network.

^n^DEWS: deep learning–based early warning system.

^o^MEWS: modified early warning system.

^p^AUPRC: area under the precision-recall curve.

^q^J48: decision tree algorithm.

^r^CC: closure classifier.

^s^MLP: multilayer perceptron.

^t^NLP: natural language processing.

^u^CNN: convolutional neural network.

^v^ET: extra tree.

^w^RNN: recurrent neural network.

^x^NCPS: National Center for Patient Safety.

^y^XGB: extreme gradient boosting.

^z^LASSO: least absolute shrinkage and selection operator.

^aa^HIT: health information technology.

^bb^PSE: patient safety event.

^cc^PANDIT: Patient Assisting Net-Based Diabetes Insulin Titration.

^dd^CHAID: Chi square automatic interaction detector.

^ee^CART: classification and regression tree.

^ff^SVR: support vector regression.

^gg^NN-BP: neural network-back propagation.

^hh^MT: model tree.

^ii^ABC4D: Advanced Bolus Calculator For Diabetes.

^jj^CSS: clinical support system.

^kk^BiLSTM: bi-long short-term memory neural network.

^ll^CRF-NN: conditional random field neural network.

^mm^LSTM-RNN: long short-term memory-recurrent neural network.

^nn^CRF: conditional random field neural network.

^oo^LRM: logistic regression probability model.

^pp^BNM: Bayesian network model.

^qq^BCP-NN: Bayesian confidence propagation neural network.

**Table 2:** Performance of artificial intelligence.

Reference	Best model recommended	Comparison/other models	Performance measures of the best model						
			Accuracy	AUROC^a^	Recall	Specificity	Precision	*F* measure	other
Huanget al [107]	SVM^b^	Logistic regression	0.675	0.771	0.632	0.789	N/A^c^	N/A	N/A
Lian et al [106]	Ensemble of three models	Bayesian network model; likelihood ratio model; BCPNN^d^	N/A	N/A	N/A	N/A	N/A	N/A	Chi-square improved by 28.83%
Chapman et al [105]	Integrated NLP^e^ with RF^f^ model for relation extraction and CRF^g^ model	CRF; RF model for relation extraction	N/A	N/A	N/A	N/A	N/A	0.612	N/A
Yang et al [104]	MADEx (long short-term memory CRF+SVM)	RNN^h^; CRF; SVM; RF	N/A	N/A	0.6542	N/A	0.5758	0.6125	N/A
Dey et al [103]	Neural fingerprint (deep learning)	10 other chemical fingerprints	0.91	0.82	0.50	0.93	N/A	0.400	N/A
Dandala et al [102]	BiLSTM^i^+CRF (joint and external resources)	BiLSTM+CRF (sequential); BiLSTM+CRF (joint)	N/A	N/A	0.822 concept extraction; 0.855 relation classification	N/A	0.846 concept extraction; 0.888 relation classification	0.83 concept extraction; 0.87 relation classification	N/A
Cai et al [101]	CARD^j^	Association rule mining	N/A	N/A	N/A	N/A	N/A	N/A	Identifying drug interaction 20%
Onay et al [100]	LSVM^k^	Boosted and bagged trees (ensemble)	0.89	0.88	0.83	1.00	N/A	0.91	N/A
Tinoco et al [99]	Computerized surveillance system	Manual chart review	N/A	N/A	N/A	N/A	N/A	N/A	Number of events detected 92% (HAI^l^), 82% (SSI^m^), 91% (LRTI^n^), 99% (UTI^o^), 100% (BSI^p^), 52% (ADE^q^)
Carrel et al [98]	NLP-assisted manual review	Manual chart review	N/A	N/A	N/A	N/A	N/A	N/A	Identified 3.1% additional patients with opioid problems
Li et al [97]	NLP-based hybrid model	Rule-based method; CRF	N/A	N/A	0.907	N/A	0.924	0.915	N/A
Schiff et al [96]	MedAware, a probabilistic machine-learning CDS^r^ system	Traditional CDS	0.75	N/A	N/A	N/A	N/A	N/A	75% of the identified alerts were clinically meaningful
Reddy et al [95]	ABC4D^s^ smartphone app (based on CBR^t^, an AI^u^ technique)	N/A	N/A	N/A	N/A	N/A	N/A	N/A	ABC4D was superior to nonadaptive bolus calculator and also more user friendly
Long et al [93]	AI smartphone app	N/A	N/A	N/A	N/A	N/A	N/A	N/A	100% adherence in the intervention group
Hasan et al [92]	Co-occurrence KNN^v^ and popular algorithm	Logistic regression; KNN; random algorithm; co-occurrence; drug popularity	N/A	N/A	N/A	N/A	N/A	N/A	Simple algorithms such as popular algorithm, co-occurrence, and KNN performed better than more complex logistic regression
Hu et al [91]	Bagged SVR^w^ and bagged voting	MLP^x^; model tree; KNN	N/A	N/A	N/A	N/A	N/A	N/A	Mean absolute error for both 0.210
Tang et al [90]	NLP	N/A	N/A	N/A	0.59	N/A	0.75	N/A	N/A
Hu et al [89]	RF	C4.5; KNN; CART^y^; MLP; logistic regression	0.839	0.912	0.782	0.888	N/A	N/A	N/A
Bean et al [88]	Own model	Logistic regression; SVM; decision tree; NLP	N/A	0.92	N/A	N/A	N/A	N/A	N/A
Hamma et al [87]	CART	CART and CHAID^z^	0.902	N/A	N/A	N/A	N/A	N/A	CHAID outperformed CART only in central nervous system classification
Song et al [86]	Similarity-based SVM	Analogous machine-learning algorithms (not mentioned)	N/A	N/A	0.24	0.97	0.68	N/A	N/A
Simon et al [85]	PANDIT^aa^	Nurses	0.635	N/A	N/A	N/A	N/A	N/A	36.5% PANDIT recommendation did not match with the nurses; 1.4% of the recommendations were unsafe.
Fong et al [84]	Unigram logistic regression	Unigram, bigram, and combined logistic regression and SVM	N/A	0.914	0.830	N/A	0.838	0.765	Unigram SVM and logistic regression were comparable
Ye et al [83]	RF	Linear and nonlinear machine-learning algorithms	N/A	N/A	N/A	N/A	N/A	N/A	C-statistic of 0.884
Marella et al [82]	Naïve Bayes kernel	Naïve Bayes; KNN and rule induction	0.855	0.927	N/A	N/A	N/A	0.877	N/A
McKnight [81]	NLP; SELF^bb^	N/A	Labeled 0.52; unlabeled 0.80	N/A	N/A	N/A	N/A	N/A	N/A
Rosenbaum and Baron [80]	SVM	Logistic regression	N/A	0.97	0.80	0.96	N/A	N/A	Positive predictive value 0.52
Wang et al [79]	Binary SVM with radial basis function kernel	Regularized logistic regression; linear SVM	N/A	N/A	0.783	N/A	0.783	0.783	N/A
Gupta and Patrick [66]	Naïve Bayes multinomial	J48; naïve Bayes; SVM	N/A	0.96	0.78	0.98	0.79	0.78	Kappa 0.76; mean absolute error 0.03
Wang et al [67]	Ensemble classifier chain of SVM with radial basis function kernel	Binary relevance of SVM, classifier chain of SVM	0.654	N/A	0.791	N/A	0.689	0.736	Hamming loss 0.80
Zhou et al [49]	SVM and RF	Naïve Bayes and MLP	N/A	N/A	0.769SVM for event type; 0.927 RF for even cause	N/A	0.788 SVM for event type; 0.927 RF for event cause	0.758 SVM for event type; 0.925 RF for event cause	N/A
Fong et al [68]	NLP with SVM	NLP with decision tree	0.990	0.960	0.920	1.00	1.000	0.960	N/A
El Messiry et al [69]	NLP	Scaled linear discriminant analysis; SVM; LASSO^cc^ and elastic-net regularized generalized linear models; max entropy; RF; neural network	0.730	N/A	0.770	0.696	N/A	N/A	N/A
Chondrogiannis et al [70]	NLP	N/A	N/A	N/A	N/A	N/A	N/A	N/A	Model developed in this study identified that each clinical report contains about 6.8 abbreviations
Liang and Gong [71]	Naïve Bayes with binary relevance	SVM; decision rule; decision tree; KNN	N/A	N/A	N/A	N/A	N/A	N/A	Micro F measure 0.212
Ong et al [72]	Text classifier with SVM	Text classifier with naïve Bayes	N/A	0.920 multitype dataset; 0.980 patient misidentification dataset	0.830 multitype dataset; 0.940 patient misidentification dataset	N/A	0.880 multitype dataset; 0.990 patient misidentification dataset	0.860 multitype dataset; 0.960 patient misidentification dataset	N/A
Taggart et al [73]	Rule-based NLP	SVM; extra trees; convolutional neural network	N/A	N/A	N/A	0.846	N/A	N/A	Positive predictive value 0.627; negative predictive value 0.971
Denecke et al [74]	AIML^dd^	N/A	N/A	N/A	N/A	N/A	N/A	N/A	Minimize information loss during clinical visits
Evans et al [75]	SVM	J48; naïve Bayes	0.728	0.891 incident type; 0.708 severity of harm	N/A	N/A	N/A	N/A	N/A
Wang et al [76]	Convolutional neural network	SVM	N/A	N/A	N/A	N/A	N/A	0.850	N/A
Klock et al [47]	SVM and RNN^ee^	RF	0.899 SVM; 0.900 RNN	N/A	N/A	N/A	N/A	0.648899 SVM; 0.889 RNN	N/A
Li et al [77]	Ensemble machine learning (bagging, boosting, and random feature method)	N/A	N/A	N/A	0.572 from 0.10 risk score; 0.855 from 0.04 risk score	N/A	N/A	N/A	C-statistic 0.880
Muff et al [78]	NLP	Patient safety indicators	N/A	N/A	0.770	0.938	N/A	N/A	N/A
Kwon et al [65]	Deep learning-based early warning system	Modified early warning system; RF; logistic regression	N/A	0.850	0.757	0.765	N/A	1.000	AUPRC^ff^
Hu et al [64]	Neural network model	ViEWS^gg^	N/A	0.880	N/A	N/A	N/A	0.81	Positive predictive value 0.726
Segal et al [63]	MedAware (a CDSS^hh^) + EHR^ii^	Legacy CDS	N/A	N/A	N/A	N/A	N/A	N/A	Clinically relevant 85%, alert burden 0.04%
Menard et al [62]	Machine learning (name not disclosed)	N/A	N/A	0.970	N/A	N/A	N/A	N/A	N/A
Eerikainen et al [61]	RF	Binary classification tree; regularized discriminant analysis classifier; SVM; RF	N/A	N/A	0.950	0.780	N/A	0.782	N/A
Antink et al [60]	Combined (selecting the best machine- learning algorithm for each alarm type)	Binary classification tree; regularized discriminant analysis classifier; SVM; RF	N/A	N/A	0.950	0.780	N/A	0.782	N/A
Zhang et al [59]	Cost-sensitive SVM	N/A	N/A	N/A	0.950	0.850	N/A	0.809	N/A
Ansari et al [58]	Multimodal machine learning using decision tree	N/A	N/A	N/A	0.890	0.850	N/A	0.762	N/A
Chen et al [57]	RF	N/A	N/A	0.870	N/A	N/A	N/A	N/A	N/A

^a^AUROC: area under the receiver operating characteristic curve.

^b^SVM: support vector machine.

^c^N/A: not applicable (Not reported).

^d^BCPNN: Bayesian confidence propagation neural network.

^e^NLP: natural language processing.

^f^RF: random forest.

^g^CRF: conditional random field.

^h^RNN: recurrent neural network.

^i^BiLSTM: Bi-long short-term memory neural network.

^j^CARD: casual association rule discovery.

^k^LSVM: linear support vector machine.

^l^HAI: hospital-associated infection.

^m^SSI: surgical site infection.

^n^LRTI: lower respiratory tract infection.

^o^UTI: urinary tract infection.

^p^BSI: bloodstream infection.

^q^ADE: adverse drug event.

^r^CDS: clinical decision support.

^s^ABC4D: Advanced Bolus Calculator For Diabetes.

^t^CBR: case-based reasoning.

^u^AI: artificial intelligence.

^v^KNN: K-nearest neighbor.

^w^SVR: support vector regression.

^x^MLP: multilayer perceptron.

^y^CART: classification and regression tree.

^z^CHAID: Chi square automatic interaction detector.

^aa^PANDIT: Patient Assisting Net-Based Diabetes Insulin Titration.

^bb^SELF: semisupervised local Fisher discriminant analysis.

^cc^LASSO: least absolute shrinkage and selection operator.

^dd^AIML: artificial intelligence markup language.

^ee^RNN: recurrent neural network.

^ff^AUPRC: area under the precision-recall curve.

^gg^VieWS: VitalPac Early Warning Score.

^hh^CDSS: clinical decision support system.

^ii^EHR: electronic health record.

### Study Themes and Findings

#### Clinical Alarms and Alerts

Nine publications addressed clinical alarms/alerts using AI techniques. The most widely used method was random forest (n=5) followed by support vector machine (n=3) and neural network/deep learning (n=3).

Studies under this category used electrocardiogram data from the PhysioNet Challenge public database and PhysioNet MIMIC II database. Five studies focused on reducing false alarm rates arising due to cardiac ailments such as arrhythmia and cardiac arrest in an intensive care unit setting [58-61,65]. The remaining four studies focused on improving the performance of clinical alarms in classifying clinical deterioration such as fluctuation in vital signs [57], predicting adverse events [62], identifying adverse medication events [63], and deterioration of patient health with hematologic malignancies [64].

#### Clinical Reports

We identified 21studies concerning clinical reports. Studies in this group primarily focused on extracting information from clinical reports such as safety reports (internal to the hospital), patient feedback, EHR notes, and others typically derived from incident monitoring systems and patient safety organizations. The most widely used method was support vector machine (n=11), followed by natural language processing (n=7) and naïve Bayes (n=5). We also identified decision trees (n=4), deep learning models (n=3), J48 (n=2), and other (n=9) algorithms.

The majority of articles focused on automating the process of patient safety classifications. These studies used machine learning and natural language processing techniques to classify clinical incidents [66] from the Incident Information Management System and to identify risky incidents [71,79,81,108] in patient safety reports retrieved from different sources, including the university database and the Veterans Affairs National Center for Patient Safety database. Some studies also analyzed medication reports [49] from structured and unstructured data obtained from the patient safety organization, and evaluated patient feedback [69] retrieved from the Patient Advocacy Reporting System developed at Vanderbilt and associated institutions.

Several studies focused on classifying the type and severity of patient safety incident reports using data collected by different sources such as universities [75], and incident reporting systems such as Advanced Incident Management Systems (across Australia) and Riskman [67,75,76]. Others analyzed hospital clinical notes internally (manually annotated by clinicians and a quality committee) and data retrieved from patient safety organizations to identify adverse incidents such as delayed medication [68], fall risks [47,67], near misses, patient misidentification, spelling errors, and ambiguity in clinical notes [109]. One study analyzed clinical study descriptions from clinicaltrials.gov and implemented an AI system to detect all abbreviations and identify their meaning to minimize incorrect interpretations [70]. Another study used inpatient laboratory test reports from Sunquest Laboratory Information System and identified wrong blood in tube errors [80].

Studies used clinical reports from various sources, including patient safety organizations, EHR data from Veterans Health Administration and Berkshire Health Systems, and deidentified notes from the Medical Information Mart for Intensive Care. These studies focused on extracting relevant information [74,77,82,84] to predict bleeding risks among critically ill patients [73], postoperative surgical complications [78], mortality risk [83], and other factors such as lab test results and vital signs [77] influencing patient safety outcomes.

#### Adverse Drug Events or Drug Safety

Twenty-three publications were classified under drug safety. These studies primarily addressed adverse effects related to drug reactions. The most widely used method was random forest (n=8), followed by natural language processing (n=7) and logistic regression (n=6). Algorithms including natural language processing (n=5), logistic regression (n=4), mobile or web apps (n=3), AI devices (n=2), and others (n=5) were also used.

Studies in this category retrieved data from different repositories such as DrugBank, Side Effect Resource, the Food and Drug Administration (FDA)’s adverse event reporting system, University of Massachusetts Medical School, Observational Medical Outcomes Partnership database, and Human Protein-Protein Interaction database to identify adverse drug interactions and reactions that can potentially negatively influence patient health [86-88,101,102,105-107,110]. Some studies also used AI to predict drug interactions by analyzing EHR data [88], unstructured discharge notes [90], and clinical charts [99,104]. One study also used AI to identify drugs that were withdrawn from the commercial markets by the FDA [100].

Some studies used AI to predict the dosage of medicines such as insulin, digoxin, and warfarin [85,89,91,95]. AI in drug safety was also used to scan through the hospital’s EHR data and identify medication errors (ie, wrong medication prescriptions) [96]. One study used AI to monitor stroke patients and track their medication (anticoagulation) intake [93]. Several studies used AI to predict a medication that a patient could be consuming but was missing from their medication list or health records [92,94,97]. Another study used AI to review clinical notes and identify evidence of opioid abuse [98].

#### Visual Representations of Safety and Chronology of the Studies

**Figure 5:**
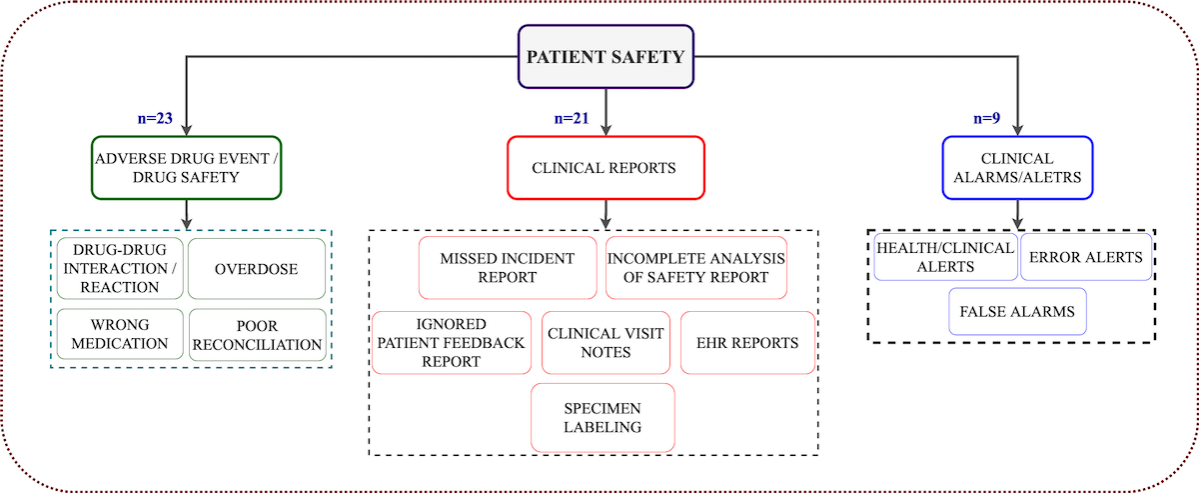
Identified factors influencing patient safety outcomes. EHR: electronic health record.

Figure 5 illustrates the details of patient safety issues/outcomes studied and reported under each classified theme using AI algorithms at the clinical level.

**Figure 6:**
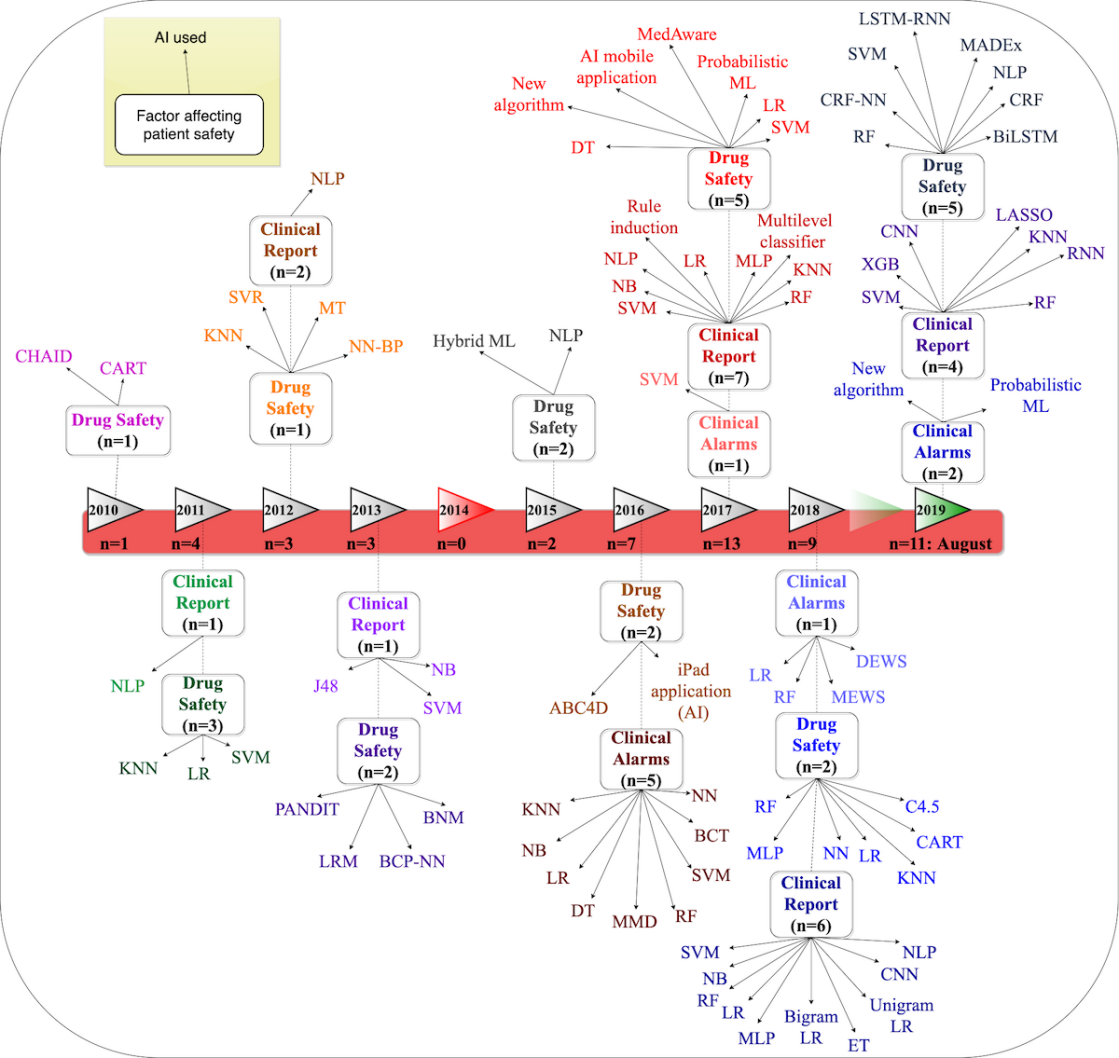
Timeline of artificial intelligence application to address factors influencing patient safety (clinical reports, drug safety, and clinical alarms) between 2009 and August 2019. ABC4D: Advanced Bolus Calculator For Diabetes; AI: artificial intelligence; BCP-NN: Bayesian confidence propagation neural network; BCT: binary classification tree; BiLSTM: bi-long short-term memory neural network; BNM: Bayesian network model; CART: classification and regression tree; CHAID: Chi-square automatic interaction detector; CRF-NN: conditional random field neural network; DEWS: deep learning-based early warning system; DT: decision tree; KNN, K-nearest neighbor; LASSO: least absolute shrinkage and selection operator; LR: logistic regression; LSTM-RNN: long short-term memory-recurrent neural network; MEWS: modified early warning system; ML: machine learning; MLP: multilayer perception; MMD; multimodal detection; MT: model tree; NB: naive Bayes; NLP: natural language processing; NN: neural network; NN-BP: neural network back propagation; PANDIT: Patient Assisting Net-Based Diabetes Insulin Titration; RF: random forest; RNN: recurrent neural network; SVM: support vector machine; SVR, support vector regression; XGB; extreme gradient boosting.

Figure 6 further shows how the application of AI in studies reporting patient safety outcomes in our review evolved over time between January 2009 and August 2019.

## Discussion

### Principal Findings

Many studies have been conducted to exhibit the analytical performance of AI in health care, particularly as a diagnostic and prognostic tool. To our knowledge, this is the first systematic review exploring and portraying studies that show the influence of AI (machine-learning and natural language processing techniques) on clinical-level patient safety outcomes. We identified 53 studies within the scope of the review. These 53 studies used 38 different types of AI systems/models to address patient safety outcomes, among which support vector machine (n=17) and natural language processing (n=12) were the most frequently used. Most of the reviewed studies reported positive changes in patient safety outcomes.

Analysis of all studies showed that there is a lack of a standardized benchmark among reported AI models. Despite varying AI performance, most studies have reported a positive impact on safety outcomes (Table 2), thus indicating that safety outcomes do not necessarily correlate to AI performance measures [26]. For example, one identified study with an accuracy of 0.63 that implemented Patient Assisting Net-Based Diabetes Insulin Titration (PANDIT) reported a negative impact of AI on safety outcomes. The PANDIT-generated recommendations that did not match with the recommendations of nurses (1.4% of the recommendations) were identified as unsafe [85]. In contrast, the study implementing natural language processing to extract clinical information from patient safety reports showed a positive impact on patient safety outcomes with accuracy of 0.53 [81]. Similarly, the FDA-approved computer-aided diagnosis of the 1990s, which significantly increased the recall rate of diagnosis, did not improve safety or patient outcomes [111]. According to our review, AI algorithms are rarely scrutinized against a standard of care (clinicians or clinical gold standard). Relying on AI outcomes that have not been evaluated against a standard benchmark that meets clinical requirements can be misleading. A study conducted in 2008 [112] developed and validated an advanced version of the QRISK cardiovascular disease risk algorithm (QRISK2). The study reported improved performance of QRISK2 when compared to its earlier version. However, QRISK2 was not compared against any clinical gold standard. Eight years later, in 2016, The Medicines & Healthcare Products Regulatory Agency identified an error in the QRISK 2 calculator [113]; QRISK2 underestimated or overestimated the potential risk of cardiovascular disease. The regulatory agency reported that a third of general practitioner surgeries in England might have been affected [113] due to the error in QRISK2. Globally, there are several Standards Development Organizations developing information technology and AI standards to address varying standardization needs in the domain of cloud computing, cybersecurity, and the internet of things [114]. However, there has been minimal effort to standardize AI in the field of health care. Health care comprises multiple departments, each having unique or different requirements (clinical standards). Thus, health care requires so-called “vertical standards,” which are standards developed for specific application areas such as drug safety (pharmaceuticals), specific surgeries, outpatients and inpatients with specific health concerns, and emergency departments [114]. In contrast, standards that are not correctly tailored for a specific purpose may hamper patient safety.

Without a standardized benchmark, it becomes challenging to evaluate whether a particular AI system meets clinical requirements (gold standard) or performs significantly better (improves patient safety) or worse (harms patient) than other similar systems in a given health care context. To generate the best possible (highest) performance outcome, AI algorithms may include unreliable confounders into the computing process. For instance, in one study, an algorithm was more likely to classify a skin lesion as malignant if an image (input data) had a ruler in it because the presence of a ruler correlated with an increased likelihood of a cancerous lesion [115]. The presence of surgical skin markings has also been shown to falsely increase a deep-learning model’s melanoma probability scores and hence the false-positive rate [116]. Moreover, there has been great emphasis focused on the importance to standardization of AI by developed countries such as the European Union, United States, China, and Japan. For instance, on February 11, 2019, the President of the United States issued an Executive Order (EO 13859) [117] directing federal agencies to actively participate in AI standards development. According to the Center for Data Innovation and The National Institute of Standards and Technology, a standardized AI benchmark can serve as a mechanism to evaluate and compare AI systems [114]. FDA Commissioner Scott Gottlieb acknowledged the importance of AI standardization that can assure that ongoing algorithm changes follow prespecified performance objectives and use a validation process that ensures safety [118].

Another major finding of this review is high heterogeneity in AI reporting. AI systems have been developed to help clinicians in estimating risks and making informed decisions. However, the evidence indicates that the quality of reporting of AI model studies is heterogeneous (not standard). Table 2 demonstrates how different studies that implemented the same AI used different evaluation metrics to measure its performance. Heterogeneity in AI reporting also makes the comparison of algorithms across studies challenging and might cause difficulties in obtaining consensus while attempting to select the best AI for a given situation. Algorithms not only need to be subjected to comparison on the same data that are representative of the target population but also the same evaluation metrics; thus, standardized reporting of AI studies would be beneficial. The current Transparent Reporting of a multivariable prediction model for Individual Prognosis Or Diagnosis (TRIPOD) consists of 22-item checklists that aim to improve the reporting of studies developing or validating a prediction model [119,120]. Studies in our review did not use TRIPOD to report findings. The possible reason behind this can be the design of TRIPOD, which focuses on a regression-based prediction model.

However, the explanation and elaboration document provides examples of good reporting methods, which are focused on models developed using regression. Therefore, a new version of the TRIPOD statement that is specific to AI/machine-learning systems (TRIPOD-ML) is in development. It will focus on the introduction of machine-learning prediction algorithms to establish methodological and reporting standards for machine-learning studies in health care [121].

Our findings also identified the need to determine the importance of an AI evaluation metric. In particular, it is important to determine which evaluation metric(s) should be measured in a given health care context. AUROC is considered to be a superior metric for classification accuracy, particularly when unbalanced datasets are used [122,123] because it is unaffected by unbalanced data, which is typical in health care. However, 36 studies in our review did not report AUROC. Evaluation measures such as precision-recall can also reflect model performance accurately [123]; however, only 11 studies in our review evaluated AI based on precision-recall. Using inappropriate measures to evaluate AI performance might impose a threat to patient safety. However, no threat to patient safety due to the use of inappropriate AI evaluation metric was identified in our review. Future studies should report the importance of evaluation metrics and determine which measure (single or multiple measures) is more important and a better representation of patient safety outcomes. More studies are needed to explore the evaluation metric(s) that should be considered before recommending an AI model.

The findings of our review demonstrate that drug safety, followed by the analysis of clinical reports, has been the most common area of interest for the use of AI to address clinical-level patient safety concerns. The wrong medication or improper dosage can result in fatal patient health outcomes and medical malpractice [91]. Of all drug safety concerns, issues related to inappropriate doses of high-alert medications are of great interest to the Joint Commission on Accreditation of Healthcare Organizations [91,124]. Medical errors are reported as the third leading cause of death in the United States. The majority of the papers in our review implemented AI to address drug safety (n=23) concerns, which is one of the most significant contributors to overall medical errors. These publications improved patient safety by identifying adverse drug reactions and preventing incorrect medications or overdoses. Future studies should further explore how to use AI systems on a larger scale to diminish medication errors at hospitals and clinics to save more lives.

Finally, the studies reviewed in this paper have addressed safety issues as identified by the Health Insurance Portability and Accountability Act (HIPAA) and the US Department of Health & Human Services (HHS). The HIPAA regulations identify risk analysis as part of the administrative safeguard requirement to improve patient safety. The HHS advocates analysis of clinical notes to track, detect, and evaluate potential risks to patients. Many studies (n=21) in our review used AI to identify patient risk from clinical notes. These studies used AI and clinical reports to extract safety-related information such as fall risks, pyxis discrepancies, patient misidentification, patient severity, and postoperative surgical complications. Our findings exhibit how, with the help of AI techniques such as natural language processing, clinical notes and reports have been used as a data source to extract patient data regarding a broad range of safety issues, including clinical notes, discharge notes, and other issues [69,70,73,84]. Our review also indicates that AI has the potential to provide valuable insights to treat patients correctly by identifying future health or safety risks [125], to improve health care quality, and reduce clinical errors [126]. Despite being recognized as one of the major factors responsible for fatigue, burnout in clinicians, and patient harm [61,127-129], only 9 studies in our review used AI to improve clinical alarms. Although studies addressing clinical alarms reported positive outcomes by minimizing false alarms and identifying patient health deterioration, the limited number of studies (n= 9) addressing these issues shows that the field is still in a nascent period of investigation. Thus, more research is needed to confirm the impact of AI on patient safety outcomes.

### Recommendations for Future Research

Future studies should work toward establishing a gold standard (for various health care contexts/ disease types/problem types) against which AI performance can be measured. Future research, as suggested by Kelly and others in 2019 [119], should also develop a common independent test (preferably for different problem types, drug safety/clinical alarms/clinical reports) using unenriched representative sample data that are not available to train algorithms.

Our review acknowledges that no single measure captures all of the desirable properties of a model, and multiple measures are typically required to summarize model performance. However, different measures are indicative of different types of analytical performance. Future studies should develop a standard framework that can guide clinicians in interpreting the clinical meaning of AI’s evaluation metrics before integrating it into the clinical workflow. Future studies should also report a quantifiable measure of AI demonstrating not only its analytical performance but also its impact on patient safety (long and short term), reliability, domain-specific risks, and uncertainty. Additionally, studies should also ensure data standardization.

Health databases or storage systems are often not compatible (integratable) across different hospitals, care providers, or different departments in the same hospital. Data in health care are largely unorganized and unstructured [9,50]. Since the performance of AI heavily depends on data, regulatory bodies should invest in data infrastructure such as standardization of EHRs and integration of different health databases. AI trained on unstructured or biased data might generate misleading results [51]. According to the National Institute of Standards and Technology (NIST), standardized data can make the training data (machine learning input) more visible and usable to authorized users. It can also ensure data quality and improve AI performance.

Most of the safety initiatives implemented in health care over the last decade have been focused on analyzing historical events to learn and evolve [130,131]. The same was also observed in our review. AI models were trained on past data. However, in health care, outcomes are satisfactory because providers make sensible and just-in-time adjustments according to the demands of the situation. Future work should train AI on the critical adjustments made by clinicians, so that AI can adapt to different conditions in the same manner as clinicians.

The integration of AI systems into the health system will alter the role of providers. Ideally, AI systems are expected to assist providers in making faster and more accurate decisions and to deliver personalized patient care. However, lack of appropriate knowledge of using complex AI systems and interpreting their outcome might impose a high cognitive workload on providers. Thus, the medical education system should incorporate necessary AI training for providers so that they can better understand the basic functioning of AI systems and extract clinically meaningful insight from the outcomes of AI.

### Limitation of this Review

This study encompasses publications that matched our inclusion criteria and operational definition of AI and patient safety. In addition, we limited the scope of AI to only machine learning and natural language processing at a clinical level. This review also only included studies published in English in the last 10 years.

### Conclusion

This systematic review identified critical research gaps that need attention from the scientific community. The majority of the studies in the review have not highlighted significant aspects of AI, such as (a) heterogeneity in AI reporting, (b) lack of a standardized benchmark, and (c) need to determine the importance of AI evaluation metric. The identified flaws of AI systems indicate that further research is needed, as well as the involvement of the FDA and NIST to develop a framework standardizing AI evaluation measures and set a benchmark to ensure patient safety. Thus, our review encourages the health care domain and AI developers to adopt an interdisciplinary and systems approach to study the overall impact of AI on patient safety outcomes and other contexts in health care.

## Appendix

Multimedia Appendix 1 Preferred Reporting Items for Systematic Reviews and Meta-Analysis (PRISMA) checklist.

## Abbreviations

AI: artificial intelligence
AUROC: area under the receiver operating characteristic curve
EHR: electronic health record
FDA: Food and Drug Administration
HHS: US Department of Health and Human Services
HIPAA: Health Insurance Portability and Accountability Act
MeSH: Medical Subject Headings
NIST: National Institute of Standards and Technology
PANDIT: Patient Assisting Net-Based Diabetes Insulin Titration
PRISMA: Preferred Reporting Items for Systematic Reviews and Meta-Analysis
TRIPOD: Transparent Reporting of a multivariable prediction model for Individual Prognosis Or Diagnosis
